# Deep Learning-Based Complete Coverage Path Planning With Re-Joint and Obstacle Fusion Paradigm

**DOI:** 10.3389/frobt.2022.843816

**Published:** 2022-03-22

**Authors:** Tingjun Lei, Chaomin Luo, Gene Eu Jan, Zhuming Bi

**Affiliations:** ^1^ Department of Electrical and Computer Engineering, Mississippi State University, Mississippi State, MS, United States; ^2^ Department of Electrical Engineering, National Taipei University, and Tainan National University of the Arts, Taipei, Taiwan; ^3^ Department of Civil and Mechanical Engineering, Purdue University Fort Wayne, Fort Wayne, IN, United States

**Keywords:** Deep learning-based path generation, complete coverage path planning, obstacle approximation and fusion, nature-inspired path planning, velocity-based local navigator, re-joint paradigm

## Abstract

With the introduction of autonomy into the precision agriculture process, environmental exploration, disaster response, and other fields, one of the global demands is to navigate autonomous vehicles to completely cover entire unknown environments. In the previous complete coverage path planning (CCPP) research, however, autonomous vehicles need to consider mapping, obstacle avoidance, and route planning simultaneously during operating in the workspace, which results in an extremely complicated and computationally expensive navigation system. In this study, a new framework is developed in light of a hierarchical manner with the obtained environmental information and gradually solving navigation problems layer by layer, consisting of environmental mapping, path generation, CCPP, and dynamic obstacle avoidance. The first layer based on satellite images utilizes a deep learning method to generate the CCPP trajectory through the position of the autonomous vehicle. In the second layer, an obstacle fusion paradigm in the map is developed based on the unmanned aerial vehicle (UAV) onboard sensors. A nature-inspired algorithm is adopted for obstacle avoidance and CCPP re-joint. Equipped with the onboard LIDAR equipment, autonomous vehicles, in the third layer, dynamically avoid moving obstacles. Simulated experiments validate the effectiveness and robustness of the proposed framework.

## 1 Introduction

In real-world applications such as environmental exploration (Rose and Chilvers, 2018), environmental sensing (Stolfi et al., 2021) and disaster response (Carrillo-Zapata et al., 2020), and other autonomous vehicle applications such as agricultural harvesting and forest surveillance, prospecting, search and rescue vehicles, concurrent complete coverage path planning (CCPP), and mapping are needed to navigate a vehicle to cover every part of the terrain in unknown environments (Wang et al., 2019; Iqbal et al., 2020; Cèsar-Tondreau et al., 2021; Meng, 2021). In previous CCPP research, the vehicle needs to concurrently consider mapping, obstacle avoidance, and route planning intractably while traversing in a workspace, which makes the entire navigation system fairly complicated and computationally expensive (Lee et al., 2014; Poonawala and Spong, 2017; Niyaz et al., 2019; Jiang et al., 2020). Particularly, in real-time navigation, re-planning with unforeseen moving obstacles may be computationally expensive. This study proposes a new framework that tackles issues of environment mapping, path generation, CCPP, and dynamic obstacle avoidance in a hierarchical manner.

### 1.1 Related Work

For decades, CCPP has undergone extensive research, and many algorithms have emerged, such as the bio-inspired neural network (BNN) approach, the Boustrophedon Cellular Decomposition (BCD) method, and the deep reinforcement learning approach (DRL). Luo and Yang (2008) developed the bio-inspired neural network (BNN) method to navigate robots to perform CCPP while avoiding obstacles within dynamic environments in real time (Zhu et al., 2017). The robot is attracted to unscanned areas and repelled by the accomplished areas or obstacles based on the neuron activity in the BNN given by the shunting equation (Yang and Luo, 2004; Li et al., 2018). Without any prior knowledge about the environment, the next position of the robot depends on the current position of the robot and neuron activity associated with its current position (Luo et al., 2016). However, it is time- and energy-consuming for the vehicles and requires high computing resources to process fine-resolution mapping (Sun et al., 2018). Unlike the BNN approach, the boundary representation method that defines the workspace is adopted by the Boustrophedon Cellular Decomposition (BCD) method and the deep reinforcement learning approach (DRL). The BCD method is proposed by Acar and Choset (2002), which decomposes the environment into many line scan partitions and is explored through a back-and-forth path (BFP) in the same direction. The BCD is an effective CCPP method with more diverse, non-polygonal obstacles in workspace. In trapezoidal decomposition as a cell, it is covered in back-and-forth patterns. For a complex configuration space with irregular-shaped obstacles, BCD needs to construct a graph that represents the adjacency connections of the cells in the boustrophedon decomposition. Therefore, a deep leaning-based method may promote it to a more efficient CCPP method (Sünderhauf et al., 2018; Valiente et al., 2020; Rawashdeh et al., 2021). Similarly, Nasirian et al. (2021) utilized traditional graph theory to segment the workspace and proposed a deep reinforcement learning approach to solve the CCPP problem in the complex workspace. However, the most common shape of the workspace is represented by polygons. As irregular areas of non-convex polygons, they can still be decomposed into multiple convex polygons (Li et al., 2011). Thus, the representation of polygons is also adopted in this study to express most workspace that needs to be explored. Such a method simplifies the complex environments and solves the covering irregularity for vehicles (Quin et al., 2021).

Faster R-CNN originated from R-CNN, and Fast CNN uses a unified neural network (NN) for object detection shown in Figure 4A. The faster R-CNN avoids using selective search, which accelerates region selection and further reduces computational costs. The faster R-CNN detector is mainly composed of a region proposal network (RPN), which generates region proposals, and a network that uses these generated feature patches (FP) for object detection. The region of interest (ROI) pooling layer is used to resize the feature patch (RFP), finally concatenated with a set of fully connected (FC) layers in our study. The two fully connected NN layers are utilized to refine the location of the bounding box and classify the objects. Faster R-CNN effectively uses the bounding box in our studies to identify and locate vehicles and obstacles in the images. This is also applied to the map obtained from farms, search, and rescue scenes to distinguish the vehicles, machines, and human beings on the image.

**FIGURE 1:**
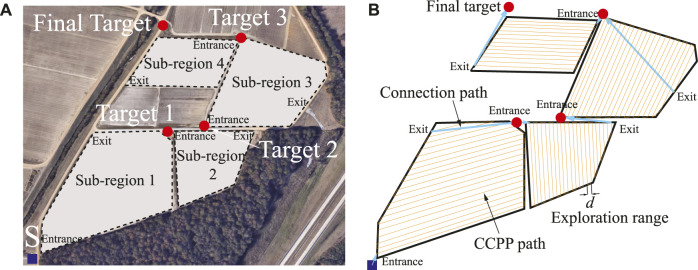
**(A)** Illustration of multiple sub-regions exploration task. **(B)** The entire CCPP trajectory of multiple sub-regions with connection paths.

Although the above-mentioned CCPP approaches have achieved remarkable results, such approaches may still be sub-optimal when the starting and target positions required by the vehicle are included in the path. Especially for multiple sub-region exploration tasks shown in Figure 1A, the task is considered continuous to explore the four sub-regions, and the starting point of the next sub-region to be explored is the target point of the last sub-region as shown by the red circles in Figure 1. The selection of intermediate target points for multiple polygonal exploration areas is still an open problem because it needs to consider the shape and relative position of each sub-region, as well as the entrance and exit of the exploration area (Graves and Chakraborty, 2018). For simplicity, the entrances of the next sub-region are selected as target points here. The connection path length from the starting point to the target point should be considered, as shown in the blue lines in Figure 1B. In this case, ignoring the connection path may increase the complete path length of the overall exploration task (Xie et al., 2019). Thus, it is vital to consider the starting and target points of the vehicle, including the exploration task, and obtain a shorter path that effectively utilizes the limited onboard resources. Another challenging problem that arises in CCPP is obstacle avoidance (An et al., 2018; Wang et al., 2021). Based on the excellent optimization and search capabilities of nature-inspired algorithms, researchers have recently explored many nature-inspired computational approaches to solve vehicle collision-free navigation problems (Deng et al., 2016; Ewerton et al., 2019; Lei et al., 2019, 2021; Segato et al., 2019). For instance, a hybrid fireworks algorithm with LIDAR-based local navigation was developed by Lei et al. (2020a), capable of generating short collision-free trajectories in unstructured environments. Zhou et al. (2019) developed a modified firefly algorithm with the self-adaptive step factor to avoid the premature and improve the operational efficiency of autonomous vehicles. Lei et al. (2020b) proposed a graph-based model integrated with ant colony optimization (ACO) to navigate the robot under the robot’s kinematics constraints. Xiong et al. (2021) further improved ACO using the time Taboo strategy to improve the algorithm convergence speed and global search ability in a dynamic environment. Cèsar-Tondreau et al. (2021) proposed a human-demonstrated navigation system, which integrates the behavioral cloning model into an off-the-shelf navigation stack.

### 1.2 Proposed Framework and Original Contributions

This study proposes a progressive three-layer framework for the CCPP navigation of autonomous vehicles. Initially, in the first layer, a new type of deep learning-based complete coverage path generation method is developed to generate complete coverage trajectories without considering obstacles. A feature learning-enabled fully convolutional deep neural network (FCNN) model is developed to identify the edges of the workspace to be explored, in combination with the starting and target positions of the vehicle to estimate waypoints given an occupancy grid map and generate the CCPP paths. The generated paths are references to guide the vehicle in the following layers to reset and continue CCPP with obstacle avoidance once traversing in the vicinity of obstacles, which improves the computational efficiency of vehicle re-planning.

In the second layer, the obstacles in the environment are considered in this stage. A nature-inspired path planning method is proposed to perform autonomous navigation of vehicles in the environment. Particularly, the vehicle deeply re-plans when it traverses in the vicinity of obstacles. In this study, the Bat algorithm is utilized to plan a collision-free trajectory in light of the size and shape of the obstacles. Once the vehicle completes the re-planning near the obstacles, a new re-joint mechanism is developed to enable the vehicle to re-join complete coverage trajectories. Additionally, an environment-based obstacle approximation and fusion paradigm is developed using image processing of feature extraction. Based on the proposed obstacle approximation and fusion method and the nature-inspired path planning method integrated with the re-joint mechanism, the autonomous vehicle takes less computational effort for optimal path planning on the map populated with obstacles.

Furthermore, a reactive local navigator in the third layer is developed to dynamically update the path and map in real time, so as to avoid moving obstacles and unknown obstacles in the dynamical environment. It dynamically adjusts the speed and direction based on onboard LIDAR sensors to navigate autonomous vehicles locally, thereby benefiting obstacle avoidance and safety assurance.

Overall, the framework composed of three layers advances accurately and is efficiently based on the environmental information layer by layer. Specifically, in the first layer, only the satellite images are needed to provide the size and shape of the searching area and the vehicle’s initial and final positions. In the second layer, the images obtained from the unmanned aerial vehicle (UAV) are required to gather detailed information of the obstacles in the environment, such as minecarts, planters, and vehicles. The third layer is based on onboard LIDAR sensors, used for real-time local reactive navigation of autonomous vehicles, avoiding moving obstacles, and building maps simultaneously. The contributions of this study are summarized as follows:

1) A hierarchical framework is proposed for the autonomous vehicle CCPP navigation in real-time environments;
2) A deep learning-based complete coverage path generation method is developed to generate complete coverage trajectories without considering obstacles;
3) For the problem of obstacle avoidance, an obstacle fusion paradigm and Bat algorithm-based path re-joint method is proposed;
4) Regarding avoiding dynamic and unknown obstacles in real-time environments, a local reactive navigator is introduced.

The rest of this study is organized as follows: in Section 2, the deep learning-based complete coverage path generation method is addressed. The second layer with regard to the nature-inspired algorithm and re-joint mechanism is explained in Section 3. Section 4 shows the reactive local navigator based on LIDAR sensors, which is the third layer in our proposed framework. Simulation and comparison studies are presented in Section 5. Several important properties of the presented framework are summarized in Section 6.

## 2 Deep Learning-Based Complete Coverage Path Planning

In the first layer, a deep learning-based method is proposed to generate a path with the starting and end positions while considering the shape of the explored areas for creating the optimal back-and-forth (BFP) coverage trajectories.

### 2.1 Preliminaries

In this section, the required assumptions are described for the proposed method. The region to be explored is assumed in a 2D environment, and the configuration space *℧* for autonomous vehicle Δ is formulated as $℧\subseteq R^{2}$ . For this study, the boundary of the area to be explored is first obtained based on image processing. There are many existing studies on edge detection (Poma et al., 2020; Nasirian et al., 2021), proving its practicability and reliability (Wagner and Oppelt, 2020). Hence, this study omitted this step and the workspace is directly analyzed. Each region is described by a standard form of convex polygon, ζ={V,E},V={1,2,…,n},E={(1,2),…,(n,1)} , where V is a set of vertices in clockwise order and E a set of edges. The vehicle’s exploration range (for the task of seeding, cleaning, rescuing, etc*.*) is a circle with a diameter of *d*. The vehicle starting position is denoted as $P_{s}$ and the end position is denoted as $P_{e}$ . The CCPP path is denoted as $\omega =\left\{F_{1},F_{2},\ldots ,F_{n}\right\}$ , while the full CCPP path is $\Omega =\left\{P_{s},\omega ,P_{e}\right\}$ . There are infinite potential solutions for covering an area known as an NP-hard problem (Arkin et al., 2000). Therefore, a variety of search patterns have been developed, such as star, zigzag, spiral, or BFP. The BFP path is utilized to establish the complete coverage path with advantages of low spatial complexity to be tracked easily by the autonomous vehicle.

### 2.2 Search Direction

**FIGURE 2:**
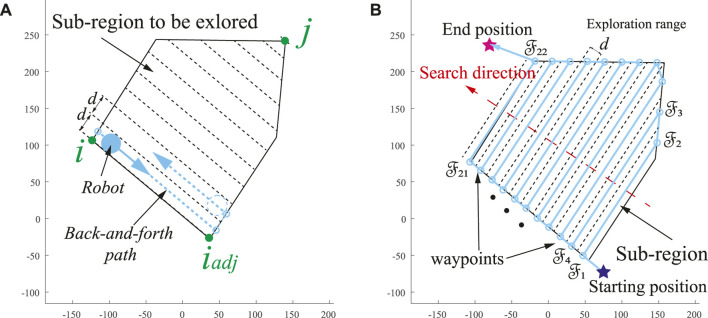
**(A)** Construction of BFP path. **(B)** BFP search direction based on the vehicle’s starting and end position.

Previous research has mainly focused on the CCPP exploration in the workspace to be explored while ignoring the vehicle’s starting and end positions in real-world scenarios. However, based on energy optimization and constraint considerations, the entire trajectories need to be considered. Therefore, for multiple edges of the polygons, the starting and end points of the vehicle should be combined to determine the vehicle’s search direction. Meanwhile, in light of the properties of the BFP-based CCPP trajectories, the optimal trajectory lines are parallel to one of the edges of the area (Torres et al., 2016). The procedure of the search direction is developed in Algorithm 1, and the process details are discussed in the following sections. The algorithm requires searching the set of opposite vertex pairs *η*, such as vertices (*i*, *j*) in Figure 2A. One of the vertexes, such as *i*, finds its adjacent vertex *i*_*adj*_, and the BFP is formed in parallel to the line $\bar{i,i_{adj}}$ with the gap distance based on the vehicle exploration range *d*. In this case, the search direction *θ* is perpendicular to $\bar{i,i_{adj}}$ toward the *j*. The function *Dist* (*i*, *j*) calculates $L_{R}$ , the total length of the BFP trajectory in the workspace.

$$L_{R}=\overset{n}{\underset{s=1}{\sum }}\sqrt{{\left(x_{F_{s+1}}-x_{F_{s}}\right)}^{2}+{\left(y_{F_{s+1}}-y_{F_{s}}\right)}^{2}}$$ (1)

Then, the starting and end points of the autonomous vehicle are enclosed in the total length L to obtain the optimal CCPP path Ω. Notably, the optimal BFP-based CCPP trajectory is first obtained in light of the edge of the explored polygon before combining it with the start and end points to obtain the minimum total length. Thus, the search direction *θ* of the BFP is obtained in the range of [ − *π*, *π*] represented by dashed lines with autonomous vehicle BFP segmentation lines, as shown in Figure 2B.

Algorithm 1 Pseudo-code for search direction.

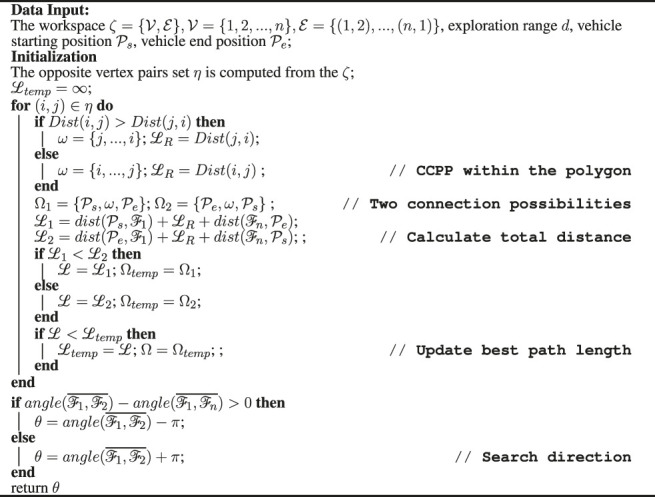

### 2.3 Deep Learning-Based Path Generation

**FIGURE 3:**
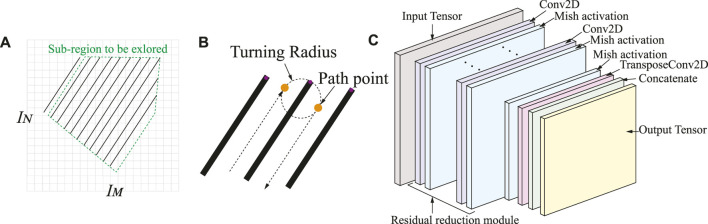
**(A)** The occupancy grid map of the workspace. **(B)** The computation of the CCPP path waypoint location based on vehicle turning radius. **(C)** Overview of the deep learning architecture.

Through the obtained BFP path segmentation line, we take the points that are intersections of the segmentation line and the workspace edge as a regression problem, and a fully convolutional deep neural network is utilized to estimate the positions of different points. In light of the turning radius of the vehicle, as shown in Figure 3B, the global CCPP trajectories are predicted by the neural network (NN) from the input image. The input image with resolution *M* × *N* is first divided into an *I*_*M*_ × *I*_*N*_ grid map (Figure 3A). The grid map *I*_*M*_ × *I*_*N*_ is *h* times smaller than the input image. Each grid contains *h* × *h* pixels, and the confidence probability C(s) denotes the confidence of the points in the grid *s*. C(s) tends to zero when no point in the grid *s* while the confidence probability C(s)>T(c) represents the possible points in the grid *s*, where T(c) denotes the confidence threshold. In each grid, the location of the final CCPP trajectory point is further refined by *δ*_*m*_ and *δ*_*n*_ in accordance with the vehicle turning radius.

The fully convolutional neural network (FCN) designed by an input tensor X(i) is gradually convolved by a stack of *n* residual reduction modules as shown in Figure 3C. Each module is composed of a series of two-dimensional convolutional layers, with Mish as the activation function, and the channel and spatial attention layer, allowing the network to highlight more relevant features. In addition, each module ends with a convolutional layer with stride 2 to reduce the spatial dimension of the input tensor. After *n* residual reduction modules, the two dimensions of the first dimension are reduced to a factor *h* + 1. Therefore, we insert a transposed convolutional layer with a stride of 2 to obtain a two-dimensional output tensor of *I*_*M*_ × *I*_*N*_ (refer to Figure 3). Add a remaining connection of the output tensor from the *n* − 1 block to include important spatial information in the tensor before the last layer. Finally, similar to a single-stage target detection network, the output tensor Y(i) and the shape *I*_*M*_ × *I*_*N*_ × 3 are calculated by 1 × 1 convolution operation, and the sigmoid and tanh are utilized as the activation of the first and last two channels, respectively. Thus, the confidence probability C(s) obtained by sigmoid predicts the existence of possible waypoints. In contrast, the tanh function is limited between −1 and +1, and the two coordinate compensations *δ*_*m*_ and *δ*_*n*_ of each unit are calculated.

## 3 Path Re-Joint and Obstacle Fusion

In the second layer, through the generated deep learning-based coverage paths, the obstacles in the environment are considered.

### 3.1 Obstacle Detection and Approximation

In the field of autonomous vehicles, map information is highly important, especially for global path planning, which determines the accuracy of the trajectory. However, for complex environments, such as disaster sites, many obstacles are scattered or gathered in various places, which bring safety and computational difficulties to the path planning of autonomous vehicles. When acquiring the disaster area or agricultural field map through drones, we need to retrieve map information to obtain specific locations of obstacles and approximate and merge a large number of obstacles into several large convex obstacles, thereby improving the efficiency of search and exploration tasks. Especially at the disaster site, the rescue time is limited, and it is important to quickly locate and approximate obstacles in the complex environment. Therefore, this section proposes an effective method for obstacle detection, obstacle approximation, and fusion.

#### 3.1.1 Object Detection With Bounding Box

Many methods have been developed for object detection. The most commonly used methods include single shot detector (SSD), region-based faster convolutional neural network (Faster R-CNN), region-based fully connected network (RCF). When these deep learning CNN models perform object detection and classification, they will obtain a bounding box based on the object’s shape. The bounding box provides us with the specific location of the object in the image and the object classification to be found. In this study, we use Faster R-CNN as our object detection method, which has been proven an efficient and accurate method in many fields (Alzadjali et al., 2021).

#### 3.1.2 Obstacle Approximation and Fusion

In this section, obstacles are approximated and merged into larger convex-shaped obstacles. Through object detection, we can obtain a large amount of information in the pictures, such as inaccessible and dangerous areas. The formed map is of great assistance to the subsequent search and distribution of ground vehicles. However, excessively unorganized obstacle information on the map will cause computational costs to vehicle path planning, especially as overlapping obstacles and excessive tiny obstacles, which are very close to each other. Therefore, it is essential to integrate multiple tiny obstacles or overlapping obstacles into an approximation of the overall obstacle.

**FIGURE 4:**
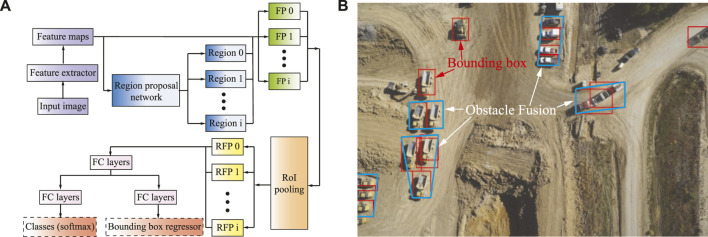
**(A)** Schematic illustration of the faster region-based convolutional neural network (Faster R-CNN) object detection method. **(B)** The obstacle approximation for a real map. Red boxes are the bounding boxes for object detection, and the green boxes are the approximation of the objects.

The method of finding the approximated obstacles is to find the obstacles to be integrated in the area. For example, in Figure 4B, the trucks parked in the mining site are considered a greater obstacle in the environment. The red bounding box is generated by the object detection method before the approximation method merges multiple bounding boxes into convex obstacles enclosed in the blue lines. Unmanned aerial vehicles (UAVs) are particularly suitable for searching large-scale farms and dangerous areas (Hassler and Baysal-Gurel, 2019). The detailed information of the scene generated through the photos of the drone’s onboard camera has made great contributions to agriculture, search, and rescue (Alzadjali et al., 2021). By merging a large number of scattered or even overlapping bounding boxes in the image to approximate as a convex obstacle, we need to select a set of suitable points from the bounding box.

**FIGURE 5:**
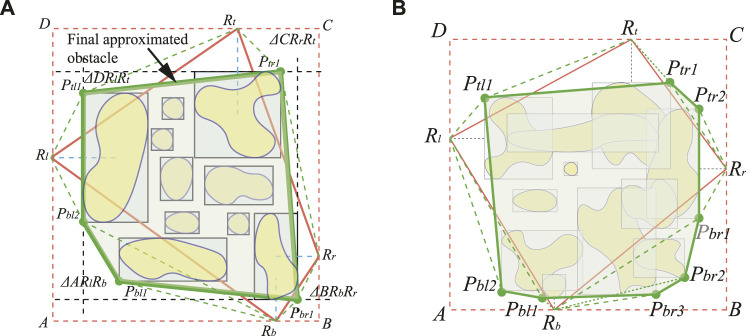
The illustration of obstacle fusion. **(A)** The final obstacle fusion for a set of non-overlapping obstacles. **(B)** The obstacle fusion for a set of overlapping obstacles\enleadertwodots.

Assuming that the image has been gathered and recognized, the selection of points that approximates the obstacles will not be internal of the bounding box; thus. only the corners of the bounding box need to be considered. We then define the four reference points as the leftmost $\left(R_{l}\right)$ , topmost $\left(R_{t}\right)$ , rightmost $\left(R_{r}\right)$ , and bottommost $\left(R_{b}\right)$ points of the convex hull as shown in Figure 5. The reference points are found by initially identifying the boundary box of obstacles to be integrated into the area. Then, it finds the midpoint of the bounding box of the boundary obstacle and expands half of the short side of the rectangular bounding box. These four reference points are extended to an axis-aligned rectangular ABCD , where A , B , C , and D are the intersection between the vertical line through one reference point and the horizontal line through another reference point. These four reference points connection lines decompose the rectangular ABCD into four triangle sections, such as top-left triangle $\Delta DR_{l}R_{t}$ . The vertex points at the top left section, top right section, bottom right section, and bottom left section are denoted as $P_{tl}$ , $P_{tr}$ , $P_{br}$ , and $P_{bl}$ , respectively. Thus, the four reference points are also denoted as $R_{l}=P_{tl0}$ , $R_{t}=P_{tr0}$ , $R_{r}=P_{bl0}$ , and $R_{b}=P_{bl0}$ . Different *h*, *i*, *j*, and *k*, implying the different numbers of vertices are contained in the top left, top right, bottom right, and bottom left section, respectively. The structure $P_{hijk}$ to be the set of vertices consists of the convex hull, such that from $R_{l}$ to $P_{tlh}$ via a number of top left corners of the bounding boxes with *m* ≤ *h*. Thus, the $P_{hijk}$ is computed in linear time using the structures $P_{mijk}$ for *m* ≤ *h*

$$P_{hijk}=\max\left(P_{mijk}+\Delta P_{tlm}P_{tlh}R_{l}\right)$$ (2)

The initial convex polygon $R_{l}R_{r}R_{t}R_{b}$ is expanded with multiple triangles starting from the reference point $R_{l}$ and there are *O* (*logn*) structures to compute in linear time. Therefore, the algorithm runs in *O* (*nlogn*) time. Because the bounding boxes in the images may overlap, this algorithm is also applicable to overlapping bounding boxes, as shown in Figure 5B. Therefore, our obstacle approximation and fusion approach adaptively fuse the bounding boxes of the detected obstacles according to the size and shape of the vehicle to rule out the gaps that are infeasible for the vehicle to pass through. Based on the proposed obstacle approximation and fusion method and the nature-inspired path planning method integrated with the re-joint mechanism, the autonomous vehicle takes less computational effort for optimal path planning on the map populated with obstacles.

### 3.2 Bat Algorithm-Based Path Re-Joint

#### 3.2.1 Bat Algorithm

The Bat algorithm (BA) is a nature-inspired population-based meta-heuristic optimization algorithm (Yang, 2010). The search strategy of the BA is inspired by the social behavior of bats and the use of echolocation in foraging and avoiding obstacles. The echolocation process of bats is addressed as follows: 1) All bats apply echolocation to sense the distance between the current position and different sources, in which all bats can distinguish food/prey and background barriers intelligently. 2) Bats automatically adjust the wavelength and frequency of their emitted ultrasonic pulses while foraging. They fly randomly at position $X_{i}$ with speed $V_{i}$ , fixed frequency $Q_{\min}$ , and loudness $A_{0}$ and continuously adjust the pulse transmission frequency R∈[0,1] depending on the proximity to the destination. 3) The loudness of the bats varies from a minimum positive constant $A_{\min}$ to $A_{0}$ . Hence, the update rule for the *i*th bat’s frequency $Q_{i}$ , speed $V_{i}^{\tau }$ , and new solution $X_{i}^{\tau }$ at time step *τ* are provided by

$$Q_{i}=Q_{\min}+\zeta \left(Q_{\max}-Q_{\min}\right)$$ (3)

$$V_{i}^{\tau }=V_{i}^{\tau -1}+\left(X_{i}^{\tau -1}-X_{gbest}\right)Q_{i}$$ (4)

$$X_{i}^{\tau }=X_{i}^{\tau -1}+V_{i}^{\tau }$$ (5)

where *ζ* denotes a randomly generated number within the interval [0, 1] and $X_{gbest}$ represents the current global best position achieved by comparing all the positions among all the bats. Because the bats also have speed limits, the speed is bond in $\left[V_{\min},V_{\max}\right]$ , where $V_{\min}=-V_{\max}$ .

In order to achieve a balance between local search and global search capabilities, a random walk procedure is processed in local search under certain probability. The new solution $X_{new}$ to replace the original solution $X_{i}^{\tau }$ is governed by

$$X_{new}=X_{i}^{\tau }+\rho \bar{A^{\tau }}$$ (6)

where *ρ* is the scaling factor which is confined to the random walk’s step size and *ρ* ∈ [ − 1, 1] is a random number. $\bar{A^{\tau }}$ is the average loudness of all bats at time step *τ*. Because bats approach their target, the amplitude of the ultrasonic pulses decreases while the pulse rate increases; the loudness $A_{i}^{\tau +1}$ and the pulse emission rate $R_{i}^{\tau +1}$ must be updated as the iteration proceeds, which is defined as

$$A_{i}^{\tau +1}=\gamma A_{i}^{\tau }$$ (7)

$$R_{i}^{\tau +1}=R^{0}\left[1-\exp\left(-\eta \tau \right)\right]$$ (8)

where *γ* and *η* are positive constants. $A^{0}$ and $R^{0}$ are initial values of loudness and pulse rate, respectively.

#### 3.2.2 Obstacle Avoidance and Path Re-Joint

**FIGURE 6:**
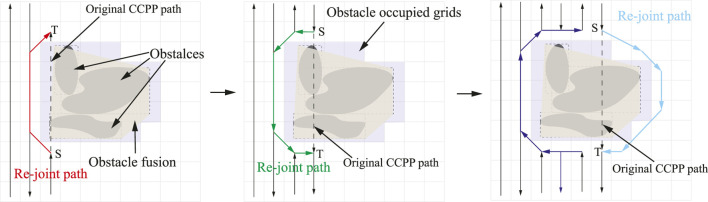
The illustration of the path re-joint mechanism.

In order to fulfill a high degree of autonomy in autonomous vehicle navigation, environment modeling or map construction is necessary to enable autonomous vehicles to generate collision-free trajectories. Therefore, in this section, the BA is utilized to perform autonomous navigation of vehicles in the grid-based environment; especially, the vehicles deeply re-plan when they traverse in the vicinity of obstacles. The grid map is composed of equal-sized grids, referred to as the generated CCPP path in Section 2. It should be noted that the grid occupied as obstacles is an inaccessible area in Figure 6. When an obstacle is presented in front of the vehicle, the current grid is defined as the initial point S , and the next point on the unoccupied grid on the CCPP path is defined as the target point T . Then, the re-joint path P is defined by the initial point S , target point T , and *n* waypoints among them:

$$P=\left[S,wp_{1},wp_{2},\ldots ,wp_{n},T\right]$$ (9)

Each point is defined by its grid coordinates (*x*, *y*), and the center of the grid pixel is regarded as a grid point. Path length is defined by the sum of the Euclidean distance between two adjacent points on the trajectory:

$$L\left(P\right)=\overset{n}{\underset{i=0}{\sum }}\sqrt{{\left(x_{wp_{i+1}}-x_{wp_{i}}\right)}^{2}+{\left(y_{wp_{i+1}}-y_{wp_{i}}\right)}^{2}}$$ (10)

where $x_{wp_{0}}$ and $x_{wp_{n+1}}$ denote starting and destination points. BA is utilized to cut down the length of point-to-point navigations. The trajectory is established between two points, which can be selected from each grid centroid of the decomposed workspace. Each point is recursively connected with the remaining points, whereas the distances of connection lines passing through obstacles are assigned with infinite numbers. As a result, the point-to-point navigations with obstacles are excluded out, and the feasible solutions are retained. The shortest paths between each pair of points are selected from those feasible solutions (Figure 6). The procedure of the proposed deep learning-based CCPP is summarized in Algorithm 2.

Algorithm 2 Procedure of proposed deep learning-based CCPP.

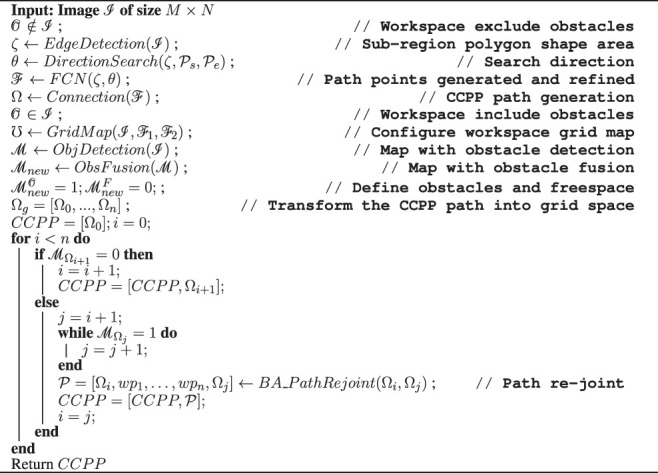

## 4 Real-Time Navigation of Autonomous Vehicles

In the third layer, once the coverage trajectories are planned, a velocity-based local reactive navigator with mapping capability is considered to avoid moving obstacles while locally constructing an environmental map. The environment of autonomous vehicle navigation is dynamic, including static obstacles and moving obstacles. The local navigation only reacts to their local environment at any moment in time, aimed to create velocity commands of an autonomous vehicle to traverse towards a destination, such as the dynamic window approach of Fox et al. (1997) and Borenstein et al. (1991). Including a sequence of bread crumbs as local waypoints in the path planning, which decomposes the coverage trajectories into a sequence of segments, makes the model particularly efficient for the environment densely populated by obstacles. In this case, a velocity obstacle approach (VOA) for real-time autonomous vehicle navigation is utilized in this study as our LIDAR-based local navigator (Fiorini and Shiller, 1998). The required information is the other sensed agents’ current position, velocity, and exact shape. The definition of the VOA is defined as follows.

**FIGURE 7:**
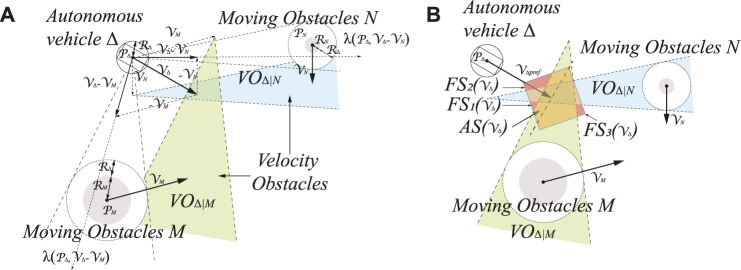
The illustration of velocity obstacle approach (VOA) in our model. **(A)** Velocity obstacles ${\mathrm{VO}}_{\Delta |M}$ and ${\mathrm{VO}}_{\Delta |N}$ for moving obstacles M and N . **(B)** Admissible velocity set (AS) and collision-free velocity set (FS) .

Δ represents the autonomous vehicle that needs to be navigated, and M and N represent the dynamic obstacles moving in the environment. Let $P_{\Delta }$ , $P_{M}$ , and $P_{N}$ denote the current positions of the autonomous vehicle *A* and dynamic obstacles M and N , respectively. Similarly, $V_{\Delta }$ , $V_{M}$ , and $V_{N}$ denote the current velocity of the autonomous vehicle Δ and dynamic obstacles M and N , respectively. The autonomous vehicle has a fixed radius $R_{\Delta }$ , a goal located at $P_{\Delta }^{goal}$ , and a preferred speed $V_{\Delta }^{pref}$ according to the road condition. To compute the velocity obstacle (VO), Δ, M and N are mapped into the configuration space, and the autonomous vehicle Δ is shrunk into a point while expanding the obstacles M and N by the radius of Δ. The VO can geometrically be interpreted in Figure 7A. It is clear that the VO of autonomous vehicle Δ caused by dynamic obstacle M , written as ${\text{VO}}_{\Delta |M}$ , is the set of all velocities of Δ resulting in a collision between Δ and M at some moment in time, assuming that M maintains its velocity $V_{M}$ . Let *P* ⊕ *Q* represent the Minkowski sum of two objects *P* and *Q*, and let − *P* represent the object *P* appearing in its reference point:

$$P⊕Q=\left\{p+q|p\in P,q\in Q\right\},-P=\left\{-p|p\in P\right\}$$ (11)

Let λ(P,V) represent a ray starting at position P and heading in the direction of velocity V :

$$\lambda \left(P,V\right)=\left\{P+tV|t\geq 0\right\}$$ (12)

As shown in Figure 7A, the $\lambda \left(P_{\Delta },V_{\Delta }-V_{M}\right)$ represents a ray starting from $P_{\Delta }$ and heading in the direction of the relative velocity of $V_{\Delta }-V_{M}$ intersecting the Minkowski sum of M and -Δ centered on $P_{M}$ . Then, velocity $V_{\Delta }$ is in the VO of M . It follows that if Δ chooses a velocity inside ${\mathrm{VO}}_{\Delta |M}$ or ${\mathrm{VO}}_{\Delta |N}$ , then Δ and M or N will collide at some point in time. If the velocity chosen is outside ${\mathrm{VO}}_{\Delta |M}$ and ${\mathrm{VO}}_{\Delta |N}$ , such a collision will never occur. Therefore, the VO of M to Δ can be represented as

$${\mathrm{VO}}_{\Delta |M}\left(V_{M}\right)=\left\{V_{\Delta }|\lambda \left(P_{\Delta },V_{\Delta }-V_{M}\right)\cap M⊕-\Delta \neq \emptyset \right\}$$ (13)

The current autonomous vehicle $V_{\Delta }$ subject to kinematics and dynamic constraints restricts the admissible set of new velocity, denoting this set as $\mathrm{AS}\left(V_{\Delta }\right)$ . According to different conditions of autonomous vehicles, such as maximum speed and maximum acceleration, $\mathrm{AS}\left(V_{\Delta }\right)$ can have any shape. In Figure 7B, an arylide yellow rectangle represents the admissible velocity set for the current velocity $V_{\Delta }$ . In each cycle of planning, the reactive navigator selects a speed that lies outside of any velocity obstacles caused through moving obstacles. As shown in Figure 7B, multiple maroon areas are collision-free velocity set $\mathrm{FS}\left(V_{\Delta }\right)$ where autonomous vehicles can avoid the moving obstacles M and N .

Our approach uses both the current position and velocity of other moving obstacles to compute their future collision-free trajectories. Obstacles are also considered in the environments, uncertainty in radius, position, and velocity, as well as dynamics and kinematics of the vehicles. The proposed velocity-based local navigator avoids unforeseen moving obstacles on the planned trajectory, which re-joins the previously planned route after it traverses in the vicinity of the obstacle. Furthermore, each layer takes advantage of the results of the previous layer as a reference to decrease the computational effort.

## 5 Simulated Experiments and Results

In this section, two simulation studies are conducted to validate the feasibility and merit of the proposed framework. The first simulation investigates the CCPP obtained by the deep learning method. The second simulation, through more detailed images obtained by drones, undertakes obstacle avoidance and re-joint paths. Moreover, onboard LIDAR is utilized to identify moving obstacles in the environment. The parameters of the proposed framework are listed below. In the CCPP deep learning training, 3000 environment maps with polygonal shape areas are utilized for training, with a resolution of 1000 × 1000 and *h* = 10. The prediction made by the NN for the image is based on the spatial dimension of 100 × 100. Parameters of BA are set as the following: $A_{0}=0.1$ , $R^{0}=0.65$ , $Q_{\min}=0.1$ , and $Q_{\max}=0.75$ . Each algorithm runs 200 times in a case, and the population size is 50.

### 5.1 Simulation and Comparative Studies in CCPP Without Obstacles

**FIGURE 8:**
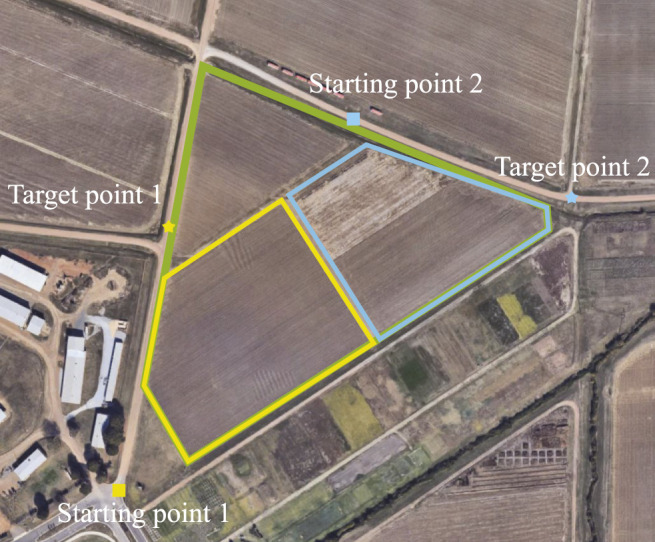
Real-world satellite images taken from Google Maps. Three targeted areas with specific starting and target points are assigned to CCPP (image from Mississippi State University North Farm).

**FIGURE 9:**
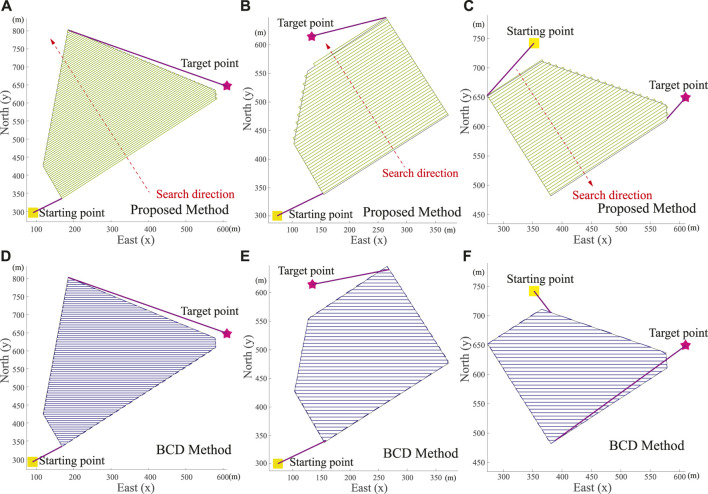
Real-world scenarios of autonomous vehicle CCPP trajectories in Figure 8. **(A–C)** Trajectories generated by proposed CCPP method. **(D–F)** Trajectories generated by Boustrophedon Cellular Decomposition (BCD) method.

In order to compare the proposed model with others, we compare the proposed CCPP method with the well-known Boustrophedon Cellular Decomposition (BCD) method (Acar and Choset, 2002). In this section, the map is a satellite image from the North Farm of Mississippi State University as shown in Figure 8. The starting and target points are randomly selected. Different shapes of the targeted areas are selected to perform coverage searches. The edges of the targeted areas as three scenarios are highlighted in yellow, blue, and green in Figure 8. The starting and target points are represented by squares and stars, respectively. The exploration range *d* of the autonomous vehicle is set as 5 m. The shape of the targeted areas, the starting and target positions of the autonomous vehicle are considered. Then, the directions of the vehicle’s search of the proposed CCPP in these three scenarios are achieved in Figures 9A–C, respectively.

In three scenarios, the proposed CCPP method has a shorter path length regardless of the coverage path within the targeted areas, the path connecting the starting and target points, and the final total path. The trajectories of the proposed CCPP method are shown in Figures 9A–C, respectively. The trajectories of the BCD method are shown in Figures 9D–F, respectively. The comparative studies are summarized in Table 1.

**TABLE 1:** Performance analysis of the proposed CCPP method with Boustrophedon Cellular Decomposition (BCD) method (Acar and Choset, 2002) under different scenarios.

Scenarios	Models	CCPP length in targeted area (m)	Connection path length (m)	Total CCPP length (m)
Figure 9A	BCD method	19 615.91	**552.38**	20 168.30
Figure 9B	Proposed method	**19 147.27**	**552.38**	**19 699.66**
Figure 9C	BCD method	8088.80	230.53	8319.33
Figure 9D	Proposed method	**7991.89**	**221.34**	**8213.23**
Figure 9E	BCD method	6579.39	315.20	6894.60
Figure 9F	Proposed method	**6480.83**	**168.58**	**6649.41**

The best results compared from two models are specified in bold.

The average precision (AP) metric is utilized to evaluate the training results. A total of 3000 synthetic images with a resolution of 800 × 800 are utilized for training and *h* = 8. Then, the network is evaluated with 1000 synthetic images. The network is trained with 200 epochs using Adam optimizer. The learning rate is equal to 3e-4, and the batch size is 16. A tunning point prediction is within the selected area as a true positive (TP), while more predictions fall within the selected range. Only one is counted as TP and all others as false positive (FP). All ground-truths not covered by a prediction are counted as false negatives (FN). Because different confidence thresholds can obtain different recall and precision values and the AP calculation is obtained by the common definition of recall and precision, we change the threshold value from 0 to 1 with step size 0.1. Multiple results obtained by modifying the threshold show that recall and precision are inversely proportional. The final confidence threshold is set as 0.9. At a distance range of 8 pixels, the average precision equals 0.9735.

**FIGURE 10:**
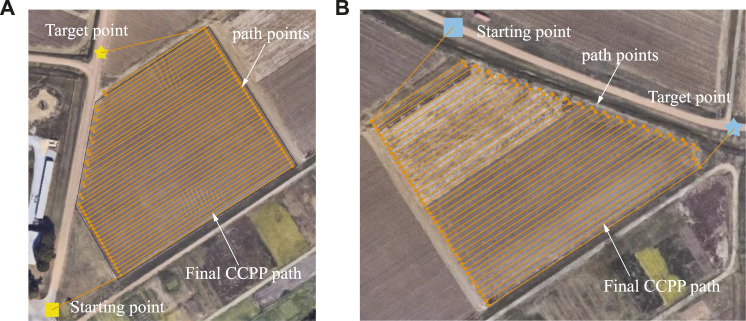
Illustration of the final trajectory in light of the deep learning-based CCPP. **(A)** The final deep learning-based CCPP path regarding the targeted area in Figure 9B. **(B)** The final deep learning-based CCPP path regarding the targeted area in Figure 9C.

Consequently, through the deep learning method, the turning points of the autonomous vehicle are generated, and the final CCPP paths are obtained, as shown in Figures 10A,B. The neural network training and testing procedure are similar to the Deepway model (Mazzia et al., 2021). However, the Deepway model relies only on identifying row-based crops to manually sort the order of waypoints that generates the final CCPP result. It remarkably limits the usage scenarios of the model and requires additional labor time to sort the waypoints. Our proposed model extends the range of usage to random environments with arbitrary shape search areas and considers the relative positions of the autonomous vehicle to obtain the optimal CCPP path, as shown in Figure 10.

### 5.2 CCPP Amid Stationary and Dynamic Obstacles

**FIGURE 11:**
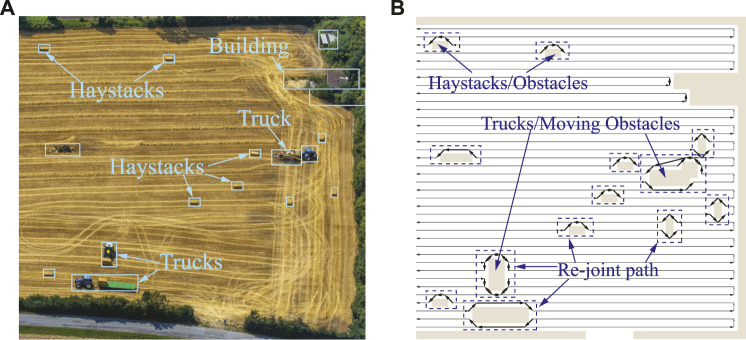
**(A)** Real-world images taken from UAVs. **(B)** Re-joint and collision-free CCPP trajectories.

**FIGURE 12:**
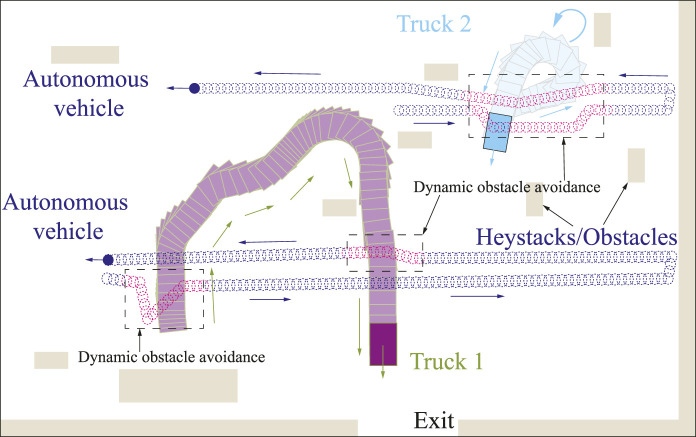
Illustration of autonomous vehicle CCPP navigation with unknown and moving obstacles in the real-world environment. The dashed boxes depict the avoidance of the moving trucks.

In this section, simulation studies are carried out to validate the second and third layers of the proposed framework, utilized for CCPP re-joint and obstacle avoidance in environments with stationary and dynamic obstacles. Due to the relatively large environment, in order to better show the re-joining path of the autonomous vehicle, a part of the map is truncated. More detailed information of the images is obtained from the drones, as shown in Figure 11A, in which haystacks and trucks are detected as obstacles. Obstacles are then approximated and merged into a grid-based map. The CCPP with obstacle avoidance function uses the CCPP path obtained in the first layer as a reference to decrease the computational effort. The proposed obstacle avoidance method based on the BA algorithm will only be triggered when an obstacle is presented in front of the vehicle. The grid of the current position is taken as the starting point, and the next grid of the CCPP reference path unoccupied by obstacles is regarded as the target point to plan a collision-free trajectory. The proposed method is unnecessarily to recalculate for the complete map, which can flexibly adapt to the alterations of obstacles in the map. The CCPP trajectory of static obstacle avoidance is shown in Figure 11B. When there are unknown and moving obstacles in the environment, such as the trucks in Figure 11, the autonomous vehicles can still rely on the onboard LIDAR to dynamically avoid obstacles and return to the original coverage path to search the entire environment. Two specific operations of the autonomous vehicle avoiding moving trucks are shown in Figure 12. The autonomous vehicle, the first truck, and the second truck are represented by dark blue circles, yellow rectangles, and light blue rectangles, respectively. The autonomous vehicle performs dynamic avoidance twice for the first truck. The vehicle successfully avoids obstacles and returns to the planned CCPP trajectory. The second truck first stops at the original position before the vehicle avoids obstacles according to the obstacle avoidance path planned by the second layer of the framework. During the returning process, the truck starts to move, and the vehicle can still avoid obstacles to the updated truck position. These results prove that the proposed CCPP framework is effective and efficient in coverage navigation under real-world applications.

## 6 Conclusion and Future Work

A new framework to tackle issues of environment mapping, path generation, CCPP, and dynamic obstacle avoidance in a hierarchical manner has been proposed. The proposed framework comprises three layers that advance more accurately and efficiently based on environmental information. The framework adopts a layer-by-layer approach with the intention of each layer treating the results of the previous layer as a reference to reduce the computational effort. Simulation studies validated the effectiveness and robustness of the proposed framework. We are working on ROS-based sensor configuration and implementation of this proposed model on an actual mobile robot. Sensors being integrated include five components: a camera, a Hokuyo LIDAR, a differential global positioning system, a digital compass, and an inertial measurement unit.

## Data Availability

The original contributions presented in the study are included in the article/supplementary material. Further inquiries can be directed to the corresponding author.
